# *De novo* biosynthesis of sterols and fatty acids in the *Trypanosoma brucei* procyclic form: Carbon source preferences and metabolic flux redistributions

**DOI:** 10.1371/journal.ppat.1007116

**Published:** 2018-05-29

**Authors:** Yoann Millerioux, Muriel Mazet, Guillaume Bouyssou, Stefan Allmann, Tiila-Riikka Kiema, Eloïse Bertiaux, Laetitia Fouillen, Chandan Thapa, Marc Biran, Nicolas Plazolles, Franziska Dittrich-Domergue, Aline Crouzols, Rik K. Wierenga, Brice Rotureau, Patrick Moreau, Frédéric Bringaud

**Affiliations:** 1 Laboratoire de Microbiologie Fondamentale et Pathogénicité (MFP), Université de Bordeaux, CNRS UMR-5234, Bordeaux, France; 2 Centre de Résonance Magnétique des Systèmes Biologiques (RMSB), Université de Bordeaux, CNRS UMR-5536, Bordeaux, France; 3 Membrane Biogenesis Laboratory, CNRS-University of Bordeaux, UMR-5200, INRA Bordeaux Aquitaine, Villenave d'Ornon, France; 4 Faculty of Biochemistry and Molecular Medicine, Biocenter Oulu, University of Oulu, Oulu, Finland; 5 Trypanosome Transmission Group, Trypanosome Cell Biology Unit, Department of Parasites and Insect Vectors, INSERM U1201, Institut Pasteur, Paris, France; 6 Metabolome Facility of Bordeaux, Functional Genomics Center, Villenave d'Ornon; University of Geneva, SWITZERLAND

## Abstract

*De novo* biosynthesis of lipids is essential for *Trypanosoma brucei*, a protist responsible for the sleeping sickness. Here, we demonstrate that the ketogenic carbon sources, threonine, acetate and glucose, are precursors for both fatty acid and sterol synthesis, while leucine only contributes to sterol production in the tsetse fly midgut stage of the parasite. Degradation of these carbon sources into lipids was investigated using a combination of reverse genetics and analysis of radio-labelled precursors incorporation into lipids. For instance, (*i*) deletion of the gene encoding isovaleryl-CoA dehydrogenase, involved in the leucine degradation pathway, abolished leucine incorporation into sterols, and (*ii*) RNAi-mediated down-regulation of the SCP2-thiolase gene expression abolished incorporation of the three ketogenic carbon sources into sterols. The SCP2-thiolase is part of a unidirectional two-step bridge between the fatty acid precursor, acetyl-CoA, and the precursor of the mevalonate pathway leading to sterol biosynthesis, 3-hydroxy-3-methylglutaryl-CoA. Metabolic flux through this bridge is increased either in the isovaleryl-CoA dehydrogenase null mutant or when the degradation of the ketogenic carbon sources is affected. We also observed a preference for fatty acids synthesis from ketogenic carbon sources, since blocking acetyl-CoA production from both glucose and threonine abolished acetate incorporation into sterols, while incorporation of acetate into fatty acids was increased. Interestingly, the growth of the isovaleryl-CoA dehydrogenase null mutant, but not that of the parental cells, is interrupted in the absence of ketogenic carbon sources, including lipids, which demonstrates the essential role of the mevalonate pathway. We concluded that procyclic trypanosomes have a strong preference for fatty acid *versus* sterol biosynthesis from ketogenic carbon sources, and as a consequence, that leucine is likely to be the main source, if not the only one, used by trypanosomes in the infected insect vector digestive tract to feed the mevalonate pathway.

## Author summary

In this study, we have (*i*) determined the carbon sources used by the *Trypanosoma brucei* procyclic insect form to feed the essential lipid biosynthetic pathways, (*ii*) further characterized the metabolic pathways leading to their degradation into acetyl-CoA (fatty acid precursor) and 3-hydroxy-3-methylglutaryl-CoA (sterol precursor) and (*iii*) showed that reduction of the ketogenic carbon sources degradation, favors their incorporation into fatty acids, instead of sterols. This fatty acid preference is compensated by an increase of leucine incorporation into sterols, which highlights the parasite adaptation capacity regarding carbon source availability by modulating the metabolic flux between branches within the network. This metabolic flexibility is particularly relevant for the insect stages of trypanosomes that evolve in the midgut and the salivary glands of their blood-feeding insect vector. One may also consider that, the metabolic flow redistribution towards the mevalonate pathway (sterol production) described *in vitro* also occurs *in vivo*, depending on the carbon source composition of the tsetse fly micro-environment, which may considerably vary along the digestive tract and depending on the fly feeding status, as well as in the other infected fly organs.

## Introduction

*Trypanosoma brucei* is a hemoparasitic unicellular eukaryote that causes Human African Trypanosomiasis (HAT), also known as sleeping sickness. The disease, fatal if untreated, is endemic in 36 countries in sub-Saharan Africa, with about 70 million people living at risk of infection [1]. The *T*. *brucei* life cycle is complex and the parasite must adapt to several dynamic micro-environments encountered both in the insect vector, tsetse fly, and in the mammalian hosts. This leads to substantial morphological and metabolic changes, including adaptation of their lipid and energy metabolism. Here, we will focus on the insect midgut procyclic stage (PCF) of the parasite by providing a comprehensive analysis of its fatty acid and sterol *de novo* biosynthesis from available carbon sources.

**Fig 1:**
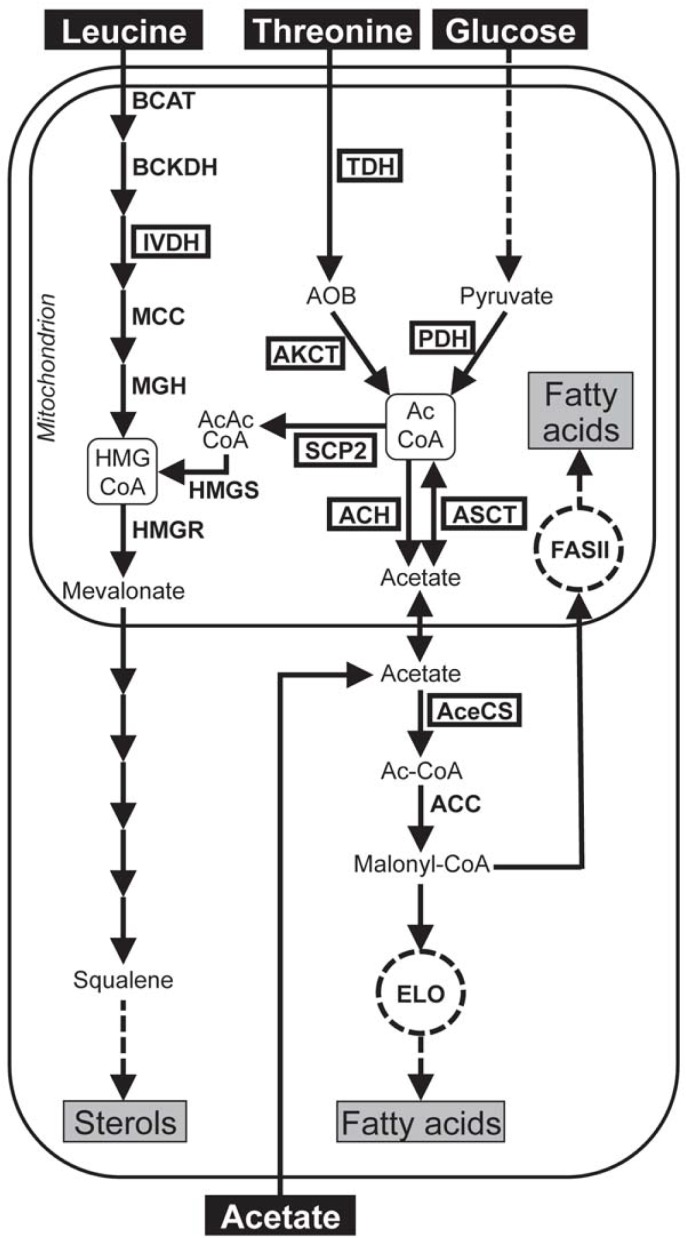
Schematic representation of fatty acid and sterol *de novo* biosynthesis in procyclic trypanosomes. Black arrows indicate enzymatic steps of leucine, glucose, threonine and acetate metabolism, with dashed arrows symbolizing several steps, to feed fatty acid and ergosterol biosynthesis. Acetyl-CoA and HMG-CoA are boxed to highlight their branching point position. For simplification and clarity only the mitochondrial subcellular compartment is represented. The microsomal elongase system and mitochondrial fatty acid synthesis are represented by a dashed circle labelled ELO and FASII, respectively. The boxed enzymes have been investigated by reverse genetics approaches in this manuscript. Abbreviations: AOB, amino oxobutyrate; Ac-CoA, acetyl-CoA; AcAc-CoA, acetoacetyl-CoA; HMG-CoA, 3-hydroxy-3-methylglutaryl-CoA. Indicated enzymes are: ACC, acetyl-CoA carboxylase; ACH, acetyl-CoA thioesterase; AceCS, AMP-dependent acetyl-CoA synthetase; AKCT, 2-amino-3-ketobutyrate CoA transferase; ASCT, acetate:succinate CoA-transferase; BCAT, branched-chain aminotransferase; BCKDH, branched-chain α-keto acid dehydrogenase complex; HMGR, HMG-CoA reductase; HMGS, HMG-CoA synthase; IVDH, isovaleryl-CoA dehydrogenase; MCC, 3-methylcrotonoyl-CoA decarboxylase; MGH, 3-methylglutaconyl-CoA hydratase; PDH, pyruvate dehydrogenase complex; SCP2, SCP2-thiolase; TDH, threonine 3-dehydrogenase.

In glucose-rich mammalian blood, the metabolism of *T*. *brucei* bloodstream forms (BSF) relies on glucose, while the poor availability of this carbohydrate in the tsetse fly midgut constrains PCF to use other carbon sources. In this context, PCF have developed an energy metabolism based on amino acids, such as proline and threonine [2, 3]. However, in the standard SDM79 glucose-rich medium, PCF preferentially utilize glucose through glycolysis [4, 5], which is converted into the excreted succinate and acetate end products [6, 7]. Acetate is synthetized into the mitochondrion from glucose and threonine-derived acetyl-CoA by two mitochondrial redundant enzymes, *i*.*e*. acetyl-CoA thioesterase (ACH, EC 3.1.2.1, Tb927.3.4260, http://www.genedb.org/genedb/tryp/) and acetate:succinate CoA transferase (ASCT, EC 2.8.3.8, Tb927.11.2690) (see Fig 1) [8, 9]. While most of the acetate is excreted by the parasite, a significant part is converted back to acetyl-CoA by the cytosolic acetyl-CoA synthetase (AceCS, EC 6.2.1.1, Tb927.8.2520) [10].

This unusual acetyl-CoA transfer system only described in trypanosomatids so far, replaces the canonical citrate shuttle required to transfer acetyl-CoA from the mitochondrion to the cytosol, which is well known in most eukaryotes. The acetate-based acetyl-CoA transfer system is essential for the parasite to feed *de novo* fatty acid biosynthesis. This is due to the cytosolic localization of the first step of both the mitochondrial type II fatty acid synthase system (FASII) and the microsomal elongase pathway (ELO), *i*.*e*. acetyl-CoA carboxylase (EC 6.4.1.2, Tb927.8.7100), which produces the malonyl-CoA precursor from acetyl-CoA [11]. Trypanosomes have developed a unique microsomal elongase pathway to produce most of their fatty acids [12, 13], whereas in other eukaryotes elongases only extend pre-existing long chain fatty acids [14]. Another specificity of *T*. *brucei* remains in the nature of the CoA primer for this elongase pathway, which depends on butyryl-CoA rather than acetyl-CoA. In addition to the ELO system, FASII contributes to approximately 10% of fatty acids by producing long chain fatty acids such as palmitate, as well as specific ones such as octanoic acid for lipoic acid biosynthesis [15, 16]. Both biosynthetic pathways are essential for growth of PCF, although trypanosomes have developed multiple ways to scavenge fatty acids present in the serum *via* uptake of protein-bound fatty acids and lysophospholipids [17].

As described for other cells, *T*. *brucei* membranes contain sterols, which regulate membrane fluidity and contribute to the organization of membrane domains. Sterols are acquired from both exogenous (lipoprotein-cholesterol endocytosis) and endogenous (*de novo* biosynthesis) sources [18]. Unlike mammalian cells but similar to fungi and *Leishmania*, trypanosomes synthetize ergosterol or ergosterol-like sterols instead of cholesterol [19]. However, the diversity of extracellular precursors and the sterol biosynthesis pathway were poorly investigated so far. *Leishmania mexicana* promastigotes use leucine as substrate to be incorporated efficiently into sterols and this pathway, even less effective, was also described in *Trypanosoma cruzi* [20, 21]. More recently, leucine has been described as a precursor for sterol biosynthesis in PCF trypanosomes [22], however, the degradation pathway leading to its integration into sterols, and its relative contribution to sterol (as well as fatty acid) *de novo* biosynthesis have not been investigated so far.

Ketogenic carbon sources degraded into acetyl-CoA can theoretically feed both fatty acid and sterol *de novo* biosynthesis in trypanosomes, as observed for glucose, threonine and acetate [23]. According to genome analyses, *T*. *brucei* lacks the enzymatic capacity to degrade other ketogenic amino acids, such as lysine, phenylalanine, tryptophan and tyrosine, into acetyl-CoA [24, 25]. However, in addition to glucose, threonine and acetate, proline is a potential lipid precursor because of its partial degradation into excreted acetate [5, 26]. In addition, the *T*. *brucei* genome contains gene candidates for all enzymes involved in degradation of isoleucine into acetyl-CoA [24, 25], although experimental evidences of its role in lipid metabolism are missing. Acetyl-CoA could also theoretically be produced by ß-oxidation of fatty acids, as reported for *Leishmania* [27, 28], however, analysis of a knock-out mutant of the single gene possibly encoding the trifunctional enzyme involved in this catabolic pathway (TFEα1, Tb927.2.4130) strongly supports the view that ß-oxidation does not occur in PCF under standard growth conditions [29].

Here, we have investigated for the first time the relative contribution of all possible carbon source candidates (proline, threonine, glucose, acetate, leucine, isoleucine and valine) to lipid production in PCF grown in rich culture medium and have characterized the 2-amino-3-ketobutyrate CoA transferase (AKCT, EC 2.3.1.29, Tb927.8.6060), which catalyses the second step of the threonine degradation pathway. We have also characterized two enzymes, isovaleryl-CoA dehydrogenase (IVDH, EC 1.3.99.10, Tb927.11.1540) and sterol carrier protein 2 thiolase (SCP2-thiolase, EC 2.3.1.9, Tb927.8.2540), involved in sterol biosynthesis from leucine or ketogenic carbon sources (glucose, threonine and acetate), respectively. Moreover, we have shown that the reverse reaction of ASCT contributes to sterol biosynthesis from acetate. We have also highlighted a metabolic redistribution in favour of fatty acid biosynthesis when acetyl-CoA production from different carbon sources is reduced. Finally, experimental infection of tsetse flies with the IVDH and AKCT null mutant parasites has further evidenced the biosynthetic pathway requirements and carbon sources availability in the midgut of the insect vector.

## Results

### Precursors used by procyclic trypanosomes for lipid biosynthesis

**Fig 2:**
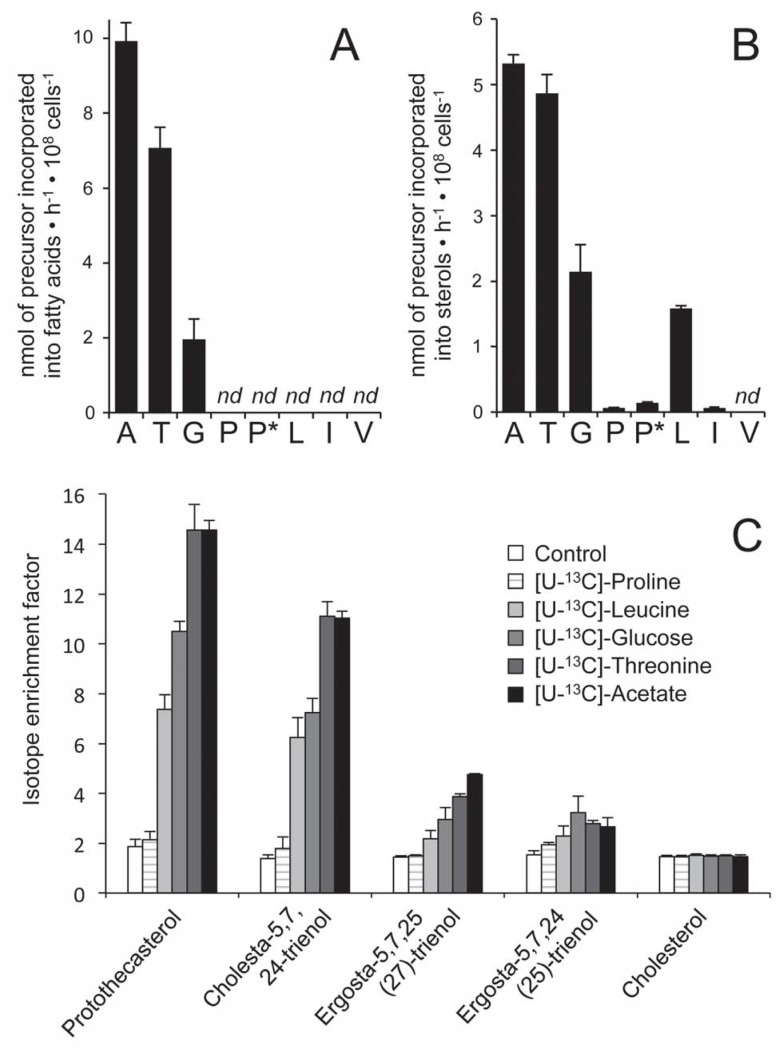
Incorporation of radio-labelled or ^13^C-labelled carbon sources into lipids of PCF trypanosomes. In panels A and B, [^14^C]-labelled fatty acid methyl esters and sterols were separated by HPTLC and analyzed as described in the Materials and methods section. Prior to lipid extraction, the EATRO1125.T7T PCF were incubated 16 h in SDM79 containing 4 mM glucose, 4 mM threonine, 4 mM proline, 4 mM leucine, 1 mM isoleucine, 1 mM valine, 4 mM acetate and one radio-labelled tracer (A, [1-^14^C]-acetate; T, L-[U-^14^C]-threonine; G, D-[U-^14^C]-glucose; P, L-[U-^14^C]-proline; L, L-[U-^14^C]-leucine; I, L-[U-^14^C]-isoleucine; V, L-[U-^14^C]-valine). The cell has also been incubated with L-[U-^14^C]-proline, 0.15 mM threonine, 1 mM isoleucine and 1 mM valine, without glucose and acetate (P*). The data are expressed as nmol of precursor (radioactive and non-radioactive molecules) incorporated into fatty acids (A) and/or sterols (B) in 10^8^ cells per hour. Error bars indicate mean ± SD of 3 biological replicates. *nd*: not detectable. In panel C, the EATRO1125.T7T PCF were incubated in the same conditions as described above with non-enriched (control) or uniformly [^13^C]-enriched proline, leucine, glucose, threonine or acetate, before processing the sample for sterol analysis by GC-MS. The isotope enrichment factor corresponds to the percentage of molecules containing ^13^C-enriched carbons. The amount of each of the five sterols identified is similar in the six experimental conditions; cholesta-5,7,24-trienol, 0.5 ± 0.1 μg; prothothecasterol, 2.2 ± 0.6 μg; ergosta-5,7,24(25)-trienol, 0.7 ± 0.1 μg; ergosta-5,7,25(27)-trienol, 2.2 ± 0.4 μg; cholesterol, 7.9 ± 1.7 μg.

Both the *de novo* biosynthesis of fatty acids and the mevalonate pathway leading to sterol production have been demonstrated to be essential for growth of the procyclic trypanosomes (PCF), however a systematic analysis of possible extracellular precursors feeding lipid biosynthesis has not been performed so far [13, 30]. To determine the carbon source preference for biosynthesis of both sterols and fatty acids, PCF were incubated overnight in SDM79 medium containing 4 mM of each carbon source analyzed (acetate, threonine, glucose, proline, leucine; except for isoleucine and valine, at 1 mM) with one of them being radio-labelled. Then, [^14^C]-labelled fatty acid methyl esters produced by transesterification and sterols were separated by HPTLC and quantified. This analysis shows that acetate and threonine are the preferred carbon sources for *de novo* synthesis of both the fatty acids and sterols, while glucose is used in a lower extent, as previously reported [23]. It is noteworthy that contribution of glucose is certainly underestimated here (up to two-fold), since expression of these data as nanomoles of glucose incorporated into sterols or fatty acids does not take into account that up to two acetyl-CoA molecules could theoretically be produced per glucose consumed [31], as opposed to acetate and threonine, which are converted into a single acetyl-CoA molecule. Proline, whose contribution to central metabolism is down-regulated in rich-medium [5], is not incorporated into fatty acids and its incorporation into sterols is close to the background level. Leucine is only a precursor of sterols and does not contribute to fatty acid biosynthesis, while incorporation of radio-labelled isoleucine into sterols is 20-fold lower compared to leucine (Fig 2A and 2B). Incorporation of [^14^C]-labelled valine, which can theoretically be converted into acetyl-CoA [24], is not detected in sterols and fatty acids.

To further characterize the *de novo* synthesized sterol and fatty acid molecules, incorporation of [^13^C]-labelled acetate, threonine, glucose, leucine and proline into lipids of PCF incubated overnight in SDM79 medium containing 4 mM of each carbon source, was determined by gas chromatography-mass spectrometry (GC-MS). This approach allowed us to estimate the incorporation of [^13^C]-enriched precursors into different lipids whose nature is determined by their absolute mass. Five sterol molecules, including cholesterol and two ergosterol derivatives (ergosta-5,7,24(25)-trienol and ergosta-5,7,25(27)-trienol), were identified by GC-MS. In the absence of [^13^C]-labelled precursors (control), less than 2% of each identified sterols were [^13^C]-enriched, which corresponds to natural ^13^C-enrichment (Fig 2C). As expected, none of the [^13^C]-labelled precursors were incorporated into cholesterol, which was taken from the medium with no detectable *de novo* biosynthesis in trypanosomatids [18]. In contrast, [^13^C]-acetate, [^13^C]-threonine, [^13^C]-glucose and [^13^C]-leucine precursors were incorporated into the other sterols, with similar incorporation profiles. This included ergosterol derivatives, which have been described as the end products of the sterol biosynthetic pathway in trypanosomatids [22]. In agreement with HPTLC data, [^13^C]-proline was not significantly incorporated into sterols (Fig 2C). However, no significant incorporation of [^13^C]-acetate and [^13^C]-glucose into fatty acids has been observed by GC-MS, in agreement with previous LC-MS/MS data [32]. This is certainly due to a combination of (*i*) the relatively high background corresponding to natural [^13^C]-enrichment (in the range of 1.5% in this series of experiments), and (*ii*) the large amount of cellular fatty acids, which are 5- to 10-fold more abundant than sterols in trypanosomes [33].

### Characterization of the complete threonine degradation pathway

**Fig 3:**
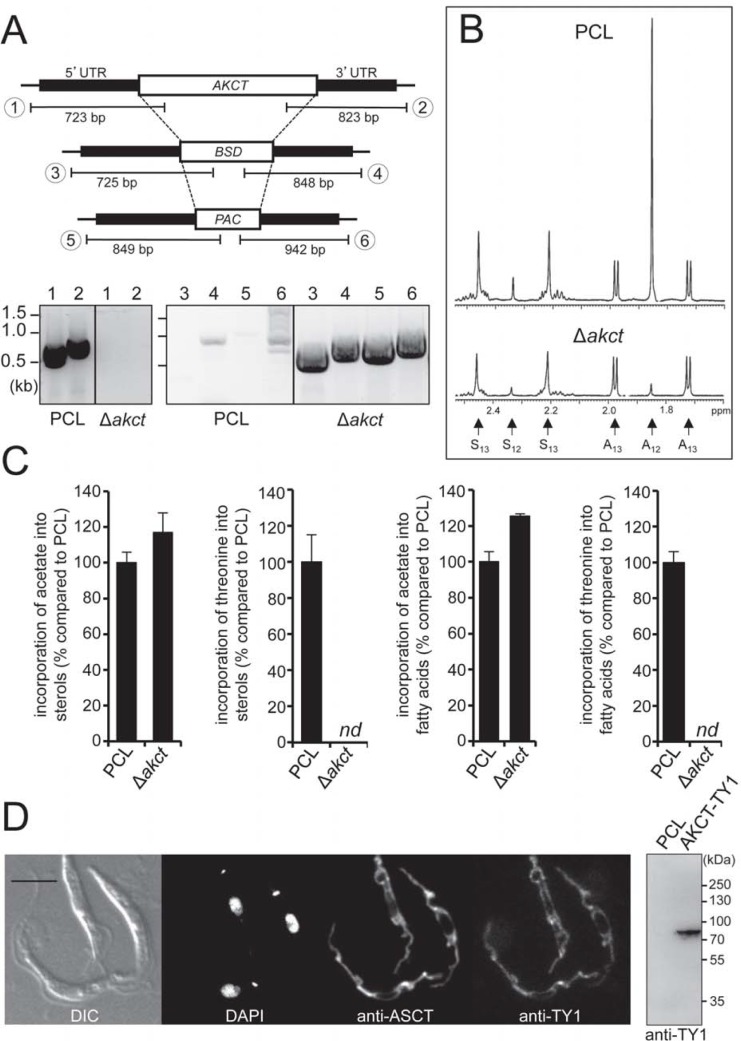
Characterization of the *AKCT* gene involved in threonine degradation. (A) PCR analysis of genomic DNA isolated from the parental EATRO1125.T7T (PCL) and Δ*akct* cells. Amplifications were performed with primers based on sequences flanking the 5’UTR and 3’UTR fragments used to target the *AKCT* gene depletion (black boxes) and primers binding the ORF of the *AKCT* gene (PCR products 1 and 2), or the blasticidin (*BSD*, PCR products 3 and 4) and the puromycin (*PAC*, PCR products 5 and 6) resistance genes. (B) NMR analysis of the EATRO1125.T7T (PCL) and Δ*akct* cell lines using D-[U-^13^C]-glucose and threonine. Excretion of ^13^C-enriched succinate (S_13_) and acetate (A_13_) and non-enriched succinate (S_12_) and acetate (A_12_) is displayed. (C) L-[U-^14^C]-threonine and [1-^14^C]-acetate incorporation into sterols and fatty acids, of parental (PCL) and Δ*akct* cell lines. [^14^C]-labelled fatty acid methyl esters and sterols were separated by HPTLC after transesterification and analyzed as described in the Materials and methods section. Error bars indicate mean ± SD of 3 biological replicates. Data are normalized with the parental cell line (PCL) values with an arbitrary value of 100 for the PCL samples. For more detail see Fig 2 legend. (D) Localization of the TY1-tagged AKCT by immunofluorescence microscopy, with the anti-ASCT immune serum used as mitochondrial marker. The right part of the panel shows a western blot of the parental (PCL) and TY1-tagged AKCT (AKCT-TY1) cell lines with the anti-TY1 immune serum. Differential interference contrast (DIC) of cells is shown to the left of the panel. Scale bar, 5 μm.

The role of the threonine dehydrogenase (TDH, EC 1.1.1.103, Tb927.6.2790) in acetate production from threonine, by catalyzing the first step of the pathway, has been previously shown [23]. Here, we have investigated the second step of the pathway (production of acetyl-CoA and glycine from 2-amino-3-ketobutyrate) catalyzed by the 2-amino-3-ketobutyrate CoA transferase (*AKCT*) potentially encoded by a single gene in the *T*. *brucei* genome (Tb927.8.6060). To confirm the role of the *AKCT* gene in acetate production, both alleles were replaced by homologous recombination with resistance markers (Fig 3A). The AKCT activity in the *AKCT* null mutant (Δ*akct*) is close to the background level (1.2 ± 2.6 nmol min^-1^ mg^-1^ of proteins) and 10-fold reduced compared to the parental cell line (12.0 ± 1.6 nmol min^-1^ mg^-1^ of proteins), while the malic enzyme (EC 1.1.1.39) activity was similar in both samples (91.0 ± 10.3 *versus* 78.4 ± 12.1 nmol min^-1^ mg^-1^ of proteins, respectively). This shows that the *AKCT* gene is responsible for the cellular AKCT activity. It is noteworthy, that the Δ*akct* cell line showed no growth delay in normal SDM79, nor in SDM79 lacking glucose and acetate. NMR spectrometry analysis of excreted end products from threonine and glucose metabolism of parental and Δ*akct* cells was performed as previously described [23]. Cells were incubated in PBS with equal amounts (4 mM) of D-[U-^13^C]-glucose and non-enriched threonine, in order to perform a quantitative analysis of threonine-derived and glucose-derived acetate production by ^1^H-NMR. [^13^C]-Acetate derived from D-[U-^13^C]-glucose (annotated A_13_ in Fig 3B) is represented by two doublets, with chemical shifts at around 2.0 ppm and 1.75 ppm, respectively, while the central resonance (1.88 ppm, annotated A_12_) corresponds to threonine-derived non-enriched acetate. Acetate production from threonine is abolished in the Δ*akct* mutant, whereas [^13^C]-acetate is still produced from D-[U-^13^C]-glucose (Fig 3B). As expected, production of [^13^C]-succinate (annotated S_13_) from D-[U-^13^C]-glucose is not affected by *AKCT* gene deletion. The residual non-enriched acetate (A_12_) and succinate (S_12_) observed in the Δ*akct* mutant (A_12_ and S_12_) and the parental cells (S_12_) is probably derived from an unknown internal carbon source [23]. Analysis of [^14^C]-labelled lipids of the mutant and Δ*akct* cell lines incubated with L-[U-^14^C]-threonine showed that incorporation of radio-labelled carbons into both sterols and fatty acids is abolished in the Δ*akct* cell line, while, incorporation of [1-^14^C]-acetate into lipids is not affected (Fig 3C). Altogether these analyses demonstrate that AKCT is involved in lipid biosynthesis from threonine through production of acetate, as previously showed for TDH [23].

To determine the subcellular localization of AKCT, we performed *in* situ tagging in the hemizygous *AKCT* knock out cell line by adding a TY1 epitope tag to the 3'-extremity of the remaining *AKCT* allele. This cell line was then used for immunofluorescence microscopy in order to validate the subcellular localisation of the AKCT protein, which co-localizes with the mitochondrial marker protein ASCT [8] (Fig 3D). The mitochondrial localization of AKCT is consistent with the prediction with a high probability (0.91) by the MitoProt program (http://ihg.gsf.de/ihg/mitoprot.html) of a mitochondrial targeting signal corresponding to the first 15 N-terminal amino acids of the protein, as well as with the presence of AKCT in previously published mitochondrial proteomes [34–36].

### IVDH is a mitochondrial enzyme involved in sterol biosynthesis from leucine

Leucine has previously been described as a precursor for sterol biosynthesis in *Leishmania* spp. and *T*. *cruzi*, as well as in PCF of *T*. *brucei* [20–22, 37]. However, the first steps leading to HMG-CoA have not been investigated in trypanosomatids so far. According to the current model, leucine is converted in the mitochondrion through a 5-step process into 3-hydroxy-3-methylglutaryl-CoA (HMG-CoA), which is located at a branching point between the different carbon sources used for sterol biosynthesis (Fig 1). To investigate leucine degradation in PCF, the third enzyme of the pathway (isovaleryl-CoA dehydrogenase, IVDH), which oxidizes isovaleryl-CoA into 3-methylcrotonyl-CoA, was functionally expressed in *Escherichia coli* to determine its activity and raise anti-IVDH antibodies. The specific activity of the his-tagged recombinant *T*. *brucei* IVDH purified to homogeneity for its substrate (isovaleryl-CoA) is 270 ± 40 nmol min^-1^ mg^-1^ of proteins, which corresponds to a rate of 0.2 conversions per active site per sec. This confirms that our candidate gene is a *bona fide* IVDH.

**Fig 4:**
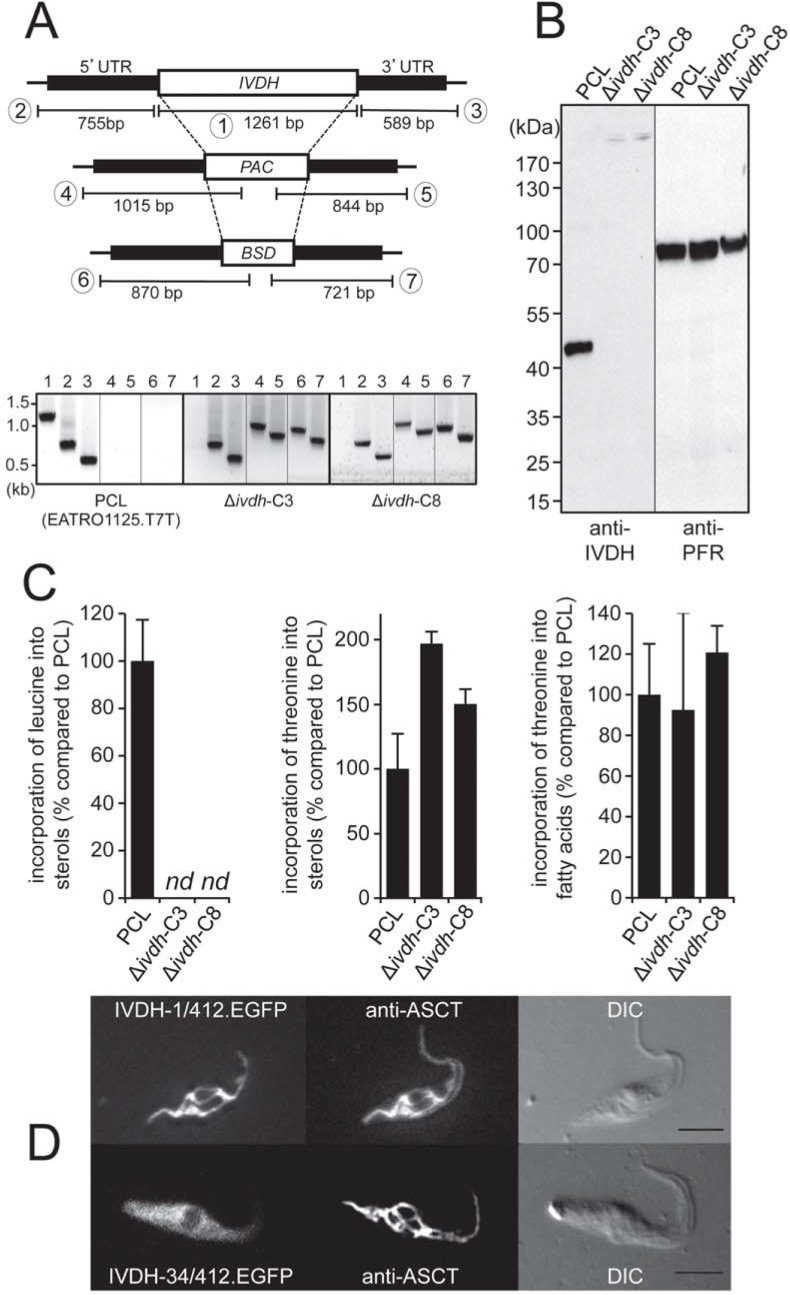
IVDH is involved in sterol biosynthesis from leucine. (A) PCR analysis of genomic DNA isolated from the parental EATRO1125.T7T (PCL), Δ*ivdh*-C3 and Δ*ivdh*-C8 cell lines. Amplifications were performed with primers based on sequences flanking the 5’UTR and 3’UTR fragments used to target the *IVDH* gene depletion (black boxes) and the 5’UTR 3’-extremity (PCR product 2), 3’UTR 5’-extremity (PCR product 3) or internal sequences of the puromycin (*PAC*, PCR products 4 and 5) and blasticidin (*BSD*, PCR products 6 and 7) resistance genes. The full-length *IVDH* coding sequence was also PCR-amplified with specific primers sequences (PCR product 1). As expected, PCR amplification using primers derived from the *IVDH* gene and drug resistant genes were only observed for the parental EATRO1125.T7T and both Δ*ivdh* cell lines, respectively. (B) Western blot analysis of the EATRO1125.T7T, Δ*ivdh*-C3 and Δ*ivdh*-C8 cell lines using anti-IVDH and anti-PFR immune sera. (C) Incorporation of [^14^C]-labelled leucine (L-[U-^14^C]-leucine) and threonine (L-[U-^14^C]-threonine) into sterols and fatty acids of parental (PCL), Δ*ivdh*-C3 and Δ*ivdh*-C8 cell lines. [^14^C]-labelled fatty acid methyl esters and sterols were separated by HPTLC after transesterification and analyzed as described in the Materials and methods section. Error bars indicate mean ± SD of 3 biological replicates. Data are normalized with the parental cell (PCL) values with an arbitrary value of 100 for the PCL samples. For more detail see Fig 2 legend. Panel D shows the mitochondrial localization of IVDH by immunofluorescence. IVDH with or without the first 33 amino acids, corresponding to the putative mitochondrial-targeting motif, was fused to the N-terminus extremity of EGFP and expressed in PCF. Fluorescence associated with EGFP is shown on the left panel and ASCT was detected with an anti-ASCT immune serum (central panel). Differential interference contrast (DIC) of cells is shown to the right of each panel. Scale bar, 5 μm.

To specifically block sterol biosynthesis from leucine, both alleles encoding *IVDH* were replaced by the BSD and PAC markers to produce the Δ*ivdh*-C3 and Δ*ivdh*-C8 cell lines. Deletion of both *IVDH* alleles was confirmed by PCR and western blotting analyses (Fig 4A and 4B). Indeed, the anti-IVDH immune serum produced against the *T*. *brucei* recombinant *IVDH* gene product (44.8 kDa) recognizes a 45 kDa protein by western blotting in the procyclic parental cells, which is no more detectable in the Δ*ivdh* cell lines. Analysis of [^14^C]-labelled sterols of the mutant and parental cell lines incubated with L-[U-^14^C]-leucine showed that incorporation of radio-labelled carbons into sterols is abolished in both Δ*ivdh* cell lines. However, incorporation of L-[U-^14^C]-threonine into fatty acids is not affected, which demonstrates that IVDH is involved in sterol biosynthesis from leucine (Fig 4C). Interestingly, incorporation of L-[U-^14^C]-threonine into sterols increased by 2- and 1.5-fold in the Δ*ivdh*-C3 and Δ*ivdh*-C8 cell lines, respectively.

The MitoProt program predicts with a high probability (0.93) that the first 33 N-terminal amino acids of IVDH constitute a mitochondrial targeting signal. The intracellular localization of IVDH was investigated by immunofluorescence microscopy of PCF expressing the full-length IVDH containing or lacking the first 33 N-terminal residues and fused with the EGFP at its C-terminal extremity (IVDH-1/412-EGFP and IVDH-34/412-EGFP, respectively). Immunofluorescence analyses revealed co-localization of IVDH-1/412-EGFP with ASCT, a known mitochondrial protein [8], while the IVDH-34/412-EGFP recombinant protein lacking the predicted mitochondrial targeting signal shows a cytosolic-like fluorescence signal (Fig 4D). These data are consistent with the presence of IVDH in previously published mitochondrial proteomes [34, 36].

### SCP2-thiolase is involved in sterol biosynthesis from glucose, threonine and acetate

**Fig 5:**
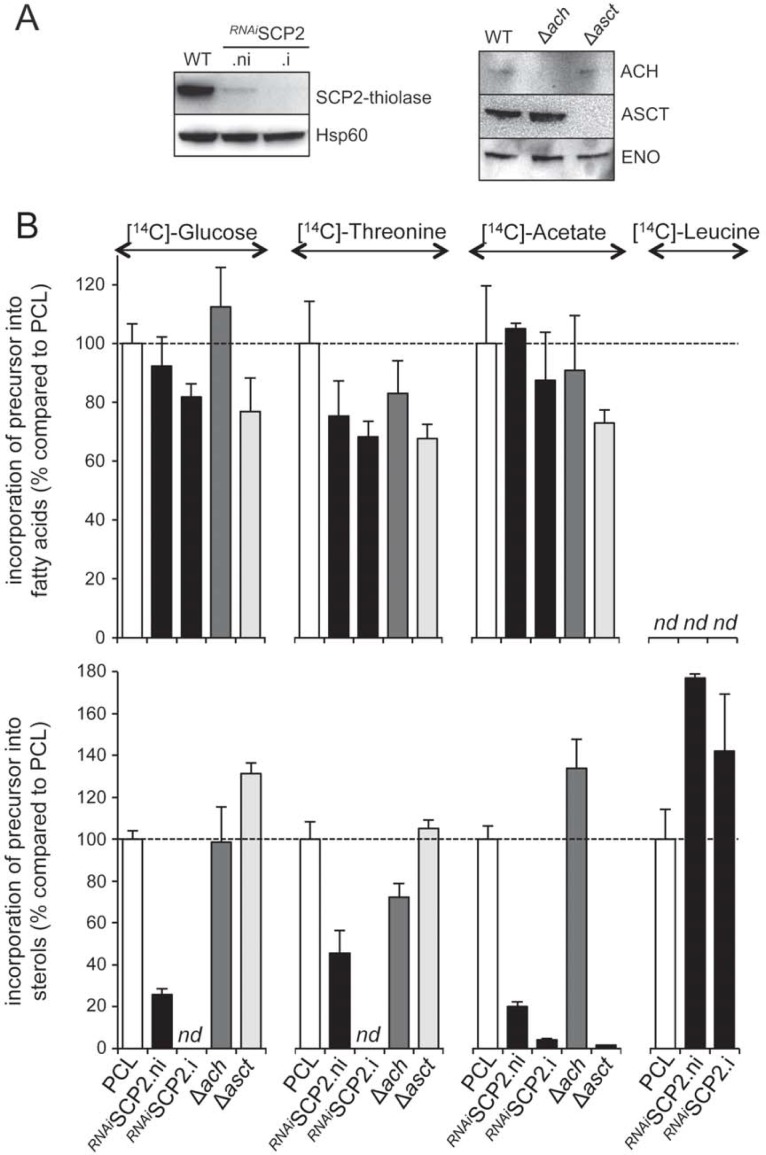
Sterol biosynthesis from glucose, threonine and acetate requires SCP2-thiolase, and fatty acid biosynthesis from acetate requires ASCT. (A) Western blot analyses of the parental (WT), knock-out and tetracycline-induced (.i) or non-induced (.ni) mutant cell lines with the immune sera indicated in the right margin. (B) The top and lower parts represent the relative incorporation of radio-labelled carbon sources (D-[U-^14^C]-glucose, L-[U-^14^C]-threonine, [1-^14^C]-acetate and L-[U-^14^C]-leucine) into fatty acids and sterols, respectively, of tetracycline-induced (.i) and non-induced (.ni) ^*RNAi*^SCP2, Δ*ach* and Δ*asct* cell lines, compared to the parental cell line (PCL). [^14^C]-labelled fatty acid methyl esters and sterols were separated by HPTLC and analyzed as described in the Materials and methods section. Data are normalized with the parental cell (PCL) values with an arbitrary value of 100 for the PCL samples, which is represented by a horizontal dashed lane. Error bars indicate mean ± SD of 3 biological replicates. *nd*: not detectable.

Glucose, threonine and acetate are precursors for biosynthesis of both fatty acids and sterols (Fig 2). The metabolic pathway leading to production of fatty acids in the endoplasmic reticulum and the mitochondrion, through the elongase system and FASII, respectively, has been described before [13, 16, 23]. However, the link between glucose-derived, threonine-derived and acetate-derived acetyl-CoA and HMG-CoA, for sterol biosynthesis, has not been investigated so far (see Fig 1). The *T*. *brucei* genome contains candidate genes for this 2-step metabolic pathway involving a thiolase (SCP2-thiolase) and HMG-CoA synthase (HMGS, EC 2.3.3.10, Tb927.8.6110) previously identified [24, 38, 39]. The *T*. *brucei* SCP2 recombinant protein, expressed and isolated from *E*. *coli*, shows thiolase activity in both the biosynthetic and degradative directions [39]. To determine the role of the SCP2-thiolase in this connecting pathway, we analyzed the impact of RNAi down-regulation of SCP2-thiolase expression (Fig 5A) on sterol and fatty acid biosynthesis from different carbon sources. As expected, fatty acid biosynthesis from threonine and glucose is not affected in the ^*RNAi*^SCP2.i cell line (Fig 5B, top panel), however, incorporation of D-[U-^14^C]-glucose, L-[U-^14^C]-threonine and [1-^14^C]-acetate into sterols is abolished in the mutant cell line (Fig 5B, lower panel). This demonstrates that SCP2-thiolase is involved in HMG-CoA production from ketogenic carbon sources, with no alternative thiolase-like activity expressed by the parasite to produce acetoacetyl-CoA from acetyl-CoA. The reduction of radiolabel incorporation into sterols in the non-induced ^*RNAi*^SCP2.ni cell line is due to the strong reduction of SCP2-thiolase expression without induction (Fig 5B). It is also noteworthy that leucine is not a precursor for fatty acid biosynthesis (Figs 2A and 5B), implying that the SCP2-thiolase/HMGS pathway is not reversible in the PCF trypanosomes.

### Sterol biosynthesis from acetate requires ASCT

Acetate is the preferred precursor for both fatty acid and sterol biosynthesis, when PCF are incubated with the same amounts of all four known precursors (Fig 2). Acetate is converted into malonyl-CoA in the cytosol to feed both the microsomal and mitochondrial fatty acid biosynthetic pathways. However, to feed sterol synthesis, acetate has to be converted into acetyl-CoA in the mitochondrion, possibly by the previously characterized ACH and/or ASCT [8, 9] (see Fig 1). To address this question, incorporation of [1-^14^C]-acetate into lipids of the previously produced Δ*ach* and Δ*asct* knock-out cell lines [9] was compared to the parental cell line. Fig 5B shows that *de novo* sterol biosynthesis from acetate is abolished in the Δ*asct* mutant, but is not affected in the Δ*ach* mutant (lower panel). As expected *de novo* fatty acid biosynthesis from acetate, glucose and threonine, as well as sterol biosynthesis from glucose and threonine are not affected in both mutant cell lines. This clearly demonstrates that mitochondrial production of acetyl-CoA from acetate solely requires ASCT, which belongs to the family I CoA-transferases previously described to catalyze reversible transfer of coenzyme A groups from CoA-thioesters to free fatty acids [40]. This also confirms that ACH irreversibly converts acetyl-CoA into acetate [9].

### Carbon flow redistribution depending on metabolism of ketogenic carbon sources

**Fig 6:**
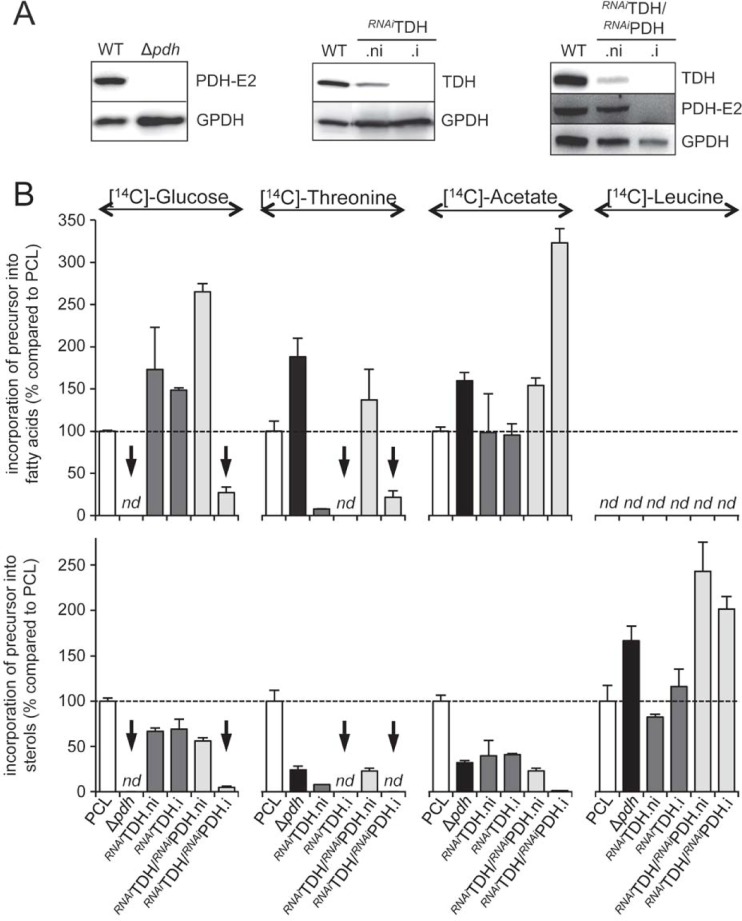
Metabolic flux redistribution between the sterol and fatty acid biosynthetic pathways. (A) Western blot analyses of the parental (WT), knock-out and tetracycline-induced (.i) or non-induced (.ni) mutant cell lines with the immune sera indicated in the right margin. (B) The top and lower parts represent the relative incorporation of radio-labelled carbon sources (D-[U-^14^C]-glucose, L-[U-^14^C]-threonine, [1-^14^C]-acetate and L-[U-^14^C]-leucine) into fatty acids and sterols, respectively, of tetracycline-induced (.i) and non-induced (.ni) ^*RNAi*^TDH, ^*RNAi*^TDH/^*RNAi*^PDH, and Δ*pdh* cell lines, compared to the parental cell line (PCL). [^14^C]-labelled fatty acid methyl esters and sterols were separated by HPTLC and analyzed as described in the Materials and mehods section. Data are normalized with the parental cell (PCL) values with an arbitrary value of 100 for the PCL samples, which is represented by a horizontal dashed lane. The arrows highlight reduction of radio-label incorporation into lipids expected from the metabolic map in Fig 1. Error bars indicate mean ± SD of 3 biological replicates. *nd*: not detectable.

Reduction of SCP2-thiolase expression in the non-induced (.ni) and induced (.i) ^*RNAi*^SCP2 cell line leads to an increase of L-[U-^14^C]-leucine incorporation into sterols, probably as a consequence of reduction/abolition of glucose-, threonine- and acetate-derived acetyl-CoA incorporation into sterols (Fig 5B, lower panel). To further study this adaptive flux redistribution, we analyzed *de novo* lipid biosynthesis in mutant cell lines affected in acetyl-CoA production from glucose and threonine. For this analysis, we have selected knock-out and/or knock-down mutant cell lines affecting expression of the E2 subunit of the pyruvate dehydrogenase complex (PDH-E2, EC 2.3.1.12, Tb927.10.7570) (Δ*pdh*), threonine dehydrogenase (TDH) (^*RNAi*^TDH) and both PDH-E2 and TDH (^*RNAi*^TDH/^*RNAi*^PDH), previously generated [23] (Fig 6A). In the same line as observed for the ^*RNAi*^SCP2 cell line, L-[U-^14^C]-leucine incorporation into sterols was increased in the ^*RNAi*^TDH/^*RNAi*^PDH mutant, probably to compensate for the reduced contribution of glucose and threonine to sterol biosynthesis. It is noteworthy that the incorporation of [1-^14^C]-acetate into fatty acids was increased by ~3-fold in the ^*RNAi*^TDH/^*RNAi*^PDH.i double mutant, certainly as a consequence of the abolition of glucose and threonine incorporation into fatty acids, with an overall incorporation of ketogenic carbon sources in fatty acid similar to the parental cell line (Fig 6B).

**Fig 7:**
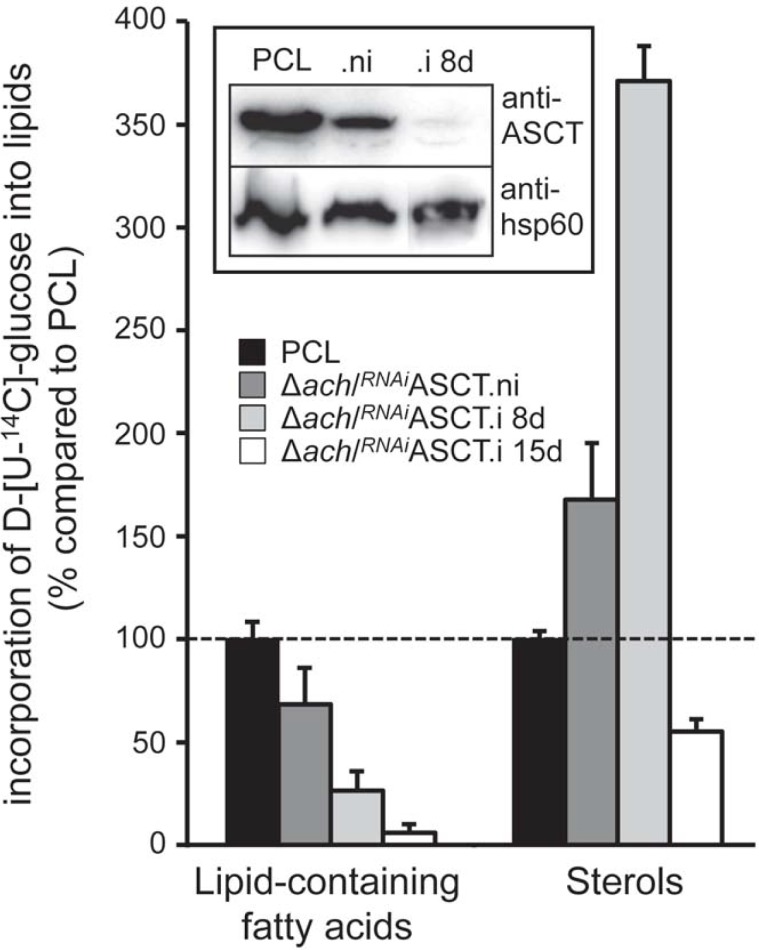
D-[U-^14^C]-glucose incorporation into lipids of the EATRO1125.T7T (PCL) and Δ*ach*/^*RNAi*^ASCT cell lines. In this experiment, trans-esterification of fatty acids was not performed before HPTLC separation and the different fatty acid-containing lipids were combined as performed before [10]. Data are normalized with the parental cell (PCL) values with an arbitrary value of 100 for the PCL samples, which is represented by a horizontal dashed lane. Black columns correspond to the EATRO1125.T7T parental cell line, grey columns to the non-induced (.ni) mutant Δ*ach*/^*RNAi*^ASCT cells, and the light grey and white columns to the mutant cells tetracycline-induced for 8 days (.i 8d) or 15 days (.i 15d), respectively. It is to note that the SDM79 used for this labelling experiment does not contain acetate. Error bars indicate mean ± SD of 3 biological replicates. The inset shows a western blot control using the anti-ASCT and anti-hsp60 immune sera.

We also investigated the fate of radio-labelled glucose incorporation into lipids, when acetyl-CoA conversion into acetate is affected in the Δ*ach*/^*RNAi*^ASCT cell line. Since growth of this cell line stops after 13 days of induction before dying two weeks later [9], the Δ*ach*/^*RNAi*^ASCT.i mutant was analyzed 8 and 15 days post-induction. As previously observed, D-[U-^14^C]-glucose incorporation into fatty acid-containing lipids is strongly reduced, however, contribution of D-[U-^14^C]-glucose to sterol biosynthesis increased 3.7-fold compared to the parental cells 8 days post-induction (Fig 7). The important reduction of D-[U-^14^C]-glucose incorporation into sterols after 15 days of induction is probably due to a general down-regulation of biosynthetic pathways consecutive of growth arrest.

In addition, distribution of glucose-, threonine- and acetate-derived acetyl-CoA between the fatty acid and sterol biosynthetic pathways, seems to be under regulation. As expected, incorporation of D-[U-^14^C]-glucose and L-[U-^14^C]-threonine into lipids (fatty acids and sterols) is abolished in the Δ*pdh* and ^*RNAi*^TDH.i cell lines, respectively (Fig 6B, indicated by an arrow). Interestingly, incorporation of radio-labelled threonine is increased into fatty acids and decreased into sterols of the Δ*pdh* mutant, while the same data are observed for the ^*RNAi*^TDH.i cell line incubated with radio-labelled glucose (Fig 6B). This suggests that the metabolic flux from these two carbon sources is redistributed towards fatty acid biosynthesis, at the expense of sterol biosynthesis. This relative flux redistribution appears clearly in the ^*RNAi*^TDH/^*RNAi*^PDH.i double mutant, which shows a 3.2-fold increase of radio-label incorporation from [1-^14^C]-acetate into fatty acids, while *de novo* synthesis of sterols from acetate is almost completely abolished (Fig 6B). Since, none of the PDH and TDH steps belong to the metabolic pathways leading to lipid biosynthesis from acetate (see Fig 1), we interpret this data as a consequence of the reduced mitochondrial acetyl-CoA pool, which is no more fed by glucose and threonine degradation. In other words, we propose that PCF trypanosomes favor fatty acid biosynthesis at the expense of sterol biosynthesis from glucose, threonine and acetate, when degradation of ketogenic carbon sources is considerably reduced.

### *In vivo* analysis of the leucine and threonine degradation pathway

**Fig 8:**
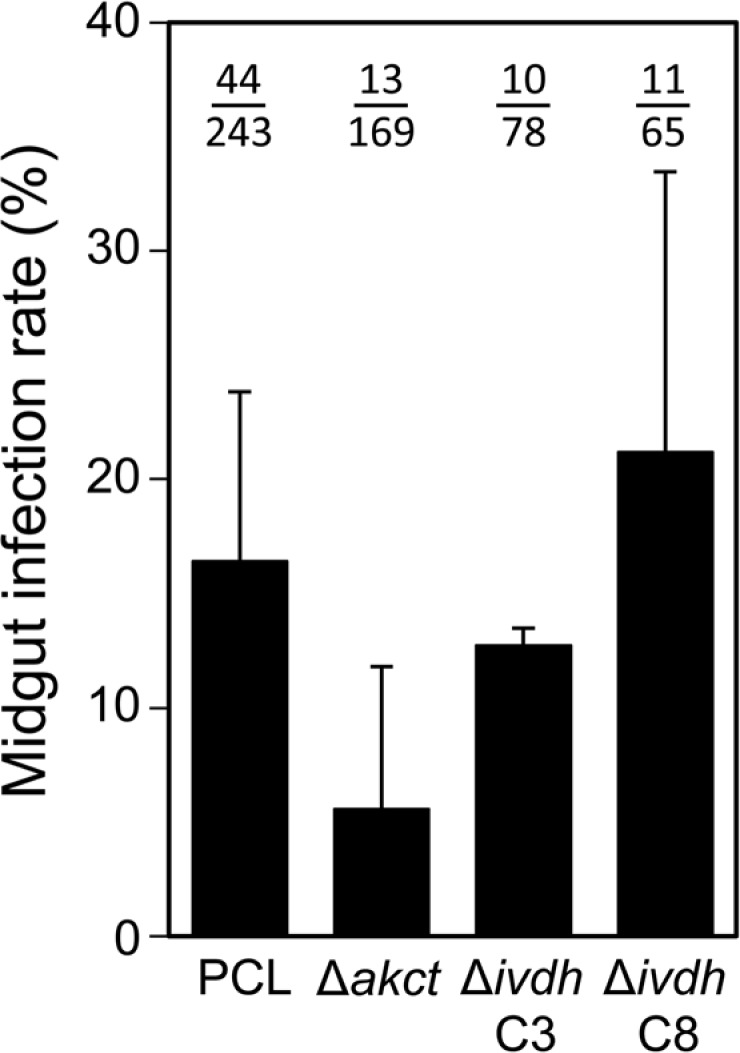
Tsetse fly midgut infection rate with the Δ*akct*, Δ*ivdh*-C3 and Δ*ivdh*-C8 cell lines. In this experiment, 50 to 100 *Glossina morsitans morsitans* teneral males were artificially fed with either the parental (PCL), Δ*akct*, Δ*ivdh*-C3 or Δ*ivdh*-C8 PCF cell lines in culture medium as previously described (n = 850) [41]. Two weeks after the infective meal, all living flies were dissected to assess the presence of parasites in their entire midgut by microscope examination. Midgut infection rates (in % ±SD) are presented for each strain as the mean of three (mutant cell line) or six (PCL) independent biological replicates (n = 243 for PCL, n = 169 for Δ*akct*, n = 78 for Δ*ivdh*-C3, n = 65 for Δ*ivdh*-C8).

We then attempted to determine whether the abolition of the incorporation of leucine and threonine in lipids had an effect on tsetse infections. A total of 850 flies (50 to 100 individuals per strain and per replicate) were fed with either the parental, Δ*akct*, Δ*ivdh*-C3 or Δ*ivdh*-C8 PCF cell lines. After 2 weeks, the midgut infection rates were 16% and 6% with the parental EATRO125.T7T and Δ*akct* procyclic cell lines, respectively, with no significant differences (Fig 8). Similarly, deletion of the *IVDH* gene did not affect the midgut infection rates with both the Δ*ivdh*-C3 and Δ*ivdh*-C8 cell lines (13% and 21%, respectively).

### IVDH is essential for PCF viability in the absence of ketogenic carbon sources, including extracellular lipids

**Fig 9:**
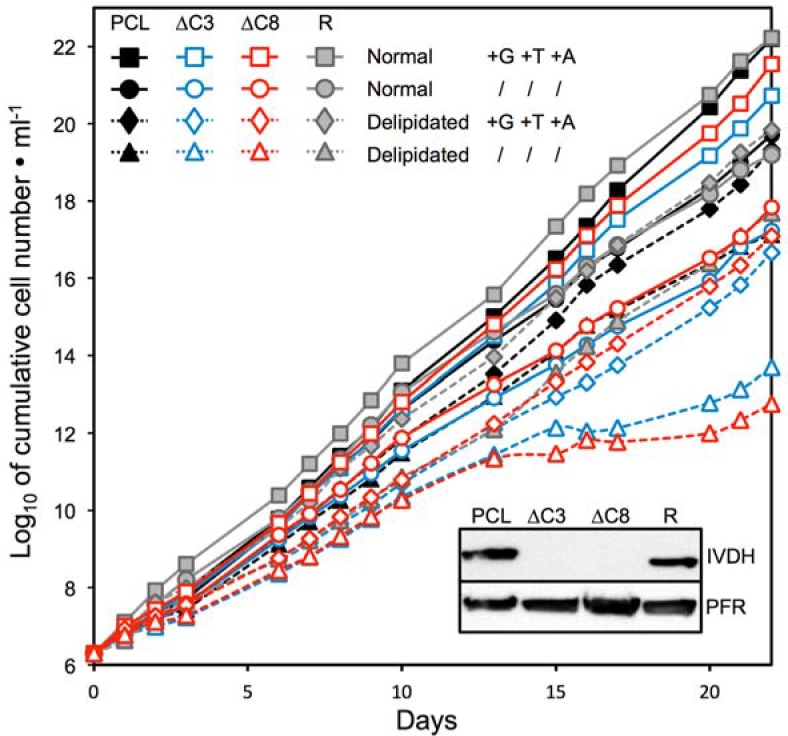
IVDH is needed for growth of PCF in medium depleted in ketogenic carbon sources. The figure shows growth curve of the parental cell line (PCL, black curve), the Δ*ivdh*-C3 (ΔC3, blue curve) and Δ*ivdh*-C8 (ΔC8, red curve) null mutants, as well as the Δ*ivdh*-C3/IVDH rescue cell line (R, grey curve) incubated in SDM79 medium containing or not 4 mM of glucose (G), threonine (T) and acetate (A), and supplemented with normal FCS (solid lines) or delipidated FCS (dashed lines). Since threonine is required for protein biosynthesis, 150 μM of this amino acid is present in the glucose/threonine/acetate-depleted conditions. Cells were maintained in the exponential growth phase (between 10^6^ and 10^7^ cells/ml) and cumulative cell numbers reflect normalization for dilution during cultivation. The inset shows western blot analysis of these cell lines with the anti-IVDH and anti-PFR immune sera.

To further address the role of IVDH in the mevalonate pathway, glucose and acetate were removed from the medium, while threonine was reduced down to 150 μM to maintain protein biosynthesis (Fig 9). The growth of the parental, Δ*ivdh*-C3 and Δ*ivdh*-C8 cell lines was not affected in the absence of these three carbon sources, suggesting either that the mevalonate pathway is not essential or that uptake of extracellular fatty acids provided by 10% fetal calf serum (FCS) can feed this pathway. Indeed, FCS contains fatty acids (free or associated with phospholipids and other lipids), which can theoretically be converted into acetyl-CoA to feed the mevalonate pathway. To address this question, growth of the Δ*ivdh* and parental cell lines were compared in SDM79 containing delipidated FCS and depleted for glucose, threonine and acetate. The parental cells were not affected, while the growth of both Δ*ivdh* mutants stopped after 15 days of incubation, which is consistent with the essential role of the mevalonate pathway (Fig 9). It is noteworthy that the observed growth recovery of the Δ*ivdh* mutants 5 days later may be due to an adaptation to the low amounts of lipids, that are only ~5-fold reduced in the commercial delipidated FCS.

To confirm this growth phenotype, an IVDH ectopic copy was re-introduced *in situ* in one *IVDH* locus of the Δ*ivdh*-C3 mutant cell line (Δ*ivdh*-C3/IVDH). The expression of IVDH was confirmed by western blotting (Fig 9, inset). As expected, growth of the Δ*ivdh*-C3/IVDH rescue cell line was restored in the delipidated SDM79 depleted for glucose, threonine and acetate (Fig 9).

## Discussion

The human and livestock pathogen *Trypanosoma brucei* has maintained and developed the ability to produce *de novo* fatty acids through the mitochondrial FASII and microsomal elongase system, as well as sterols through the mevalonate pathway, which are all essential for growth of the mammalian and insect stages of the parasite (for reviews see [16, 17, 30, 42]). Here we have performed a comprehensive metabolic analysis of all possible carbon sources feeding fatty acid and sterol biosynthesis in PCF, with the aim to complete the metabolic map, to study the regulatory interplay between the different branches of the corresponding metabolic network, and to determine the real contribution of each branch *in vivo* in the insect vector.

The *T*. *brucei* genome potentially encodes for all enzymes responsible for conversion of glucose, acetate, threonine, leucine, isoleucine, valine, proline and fatty acids into acetyl-CoA and/or HMG-CoA, the precursors for biosynthesis of fatty acids and sterols. Among them, only the four former carbon sources can support lipid biosynthesis. The absence of isoleucine and valine incorporation into lipids is consistent with previous data obtained in *Leishmania* promastigotes [20]. Proline degradation into sterols is negligible, since it solely contributes to 0.5% of sterol biosynthesis in medium containing equimolar amounts of all known precursors (Fig 2), while it is not a carbon source for fatty acid biosynthesis in PCF. These data are consistent with previous data reported for *Leishmania* promastigotes [20, 43] and with the fact that proline is primarily converted into glutamate and succinate, with barely no acetate (and acetyl-CoA) produced by PCF grown in glucose-rich conditions [5, 26]. However, PCF use proline as a main carbon source in the glucose-depleted environment of the insect midgut [3, 44]. Surprisingly, removing the main ketogenic carbon sources (glucose, threonine and acetate) from the culture medium did not significantly increase proline incorporation into lipids (Fig 2), confirming that proline-derived acetyl-CoA is not used for lipid biosynthesis. Acetyl-CoA could also theoretically be produced by ß-oxidation of fatty acids, as reported for *Leishmania* [27, 28], however, analysis of a knock-out mutant of the single gene possibly encoding for two ß-oxidation steps strongly supports the view that ß-oxidation does not occur in PCF under standard and glucose-depleted growth conditions [29]. Interestingly, the growth defect observed for the two Δ*ivdh* cell lines incubated in the glucose/threonine/acetate/lipid-depleted medium, but not in the glucose/threonine/acetate-depleted medium, suggest that ß-oxidation of fatty acids may contribute to feed the mevalonate pathway in the absence of the other ketogenic carbon sources. These preliminary data suggest that ß-oxidation occurs in PCF grown in the presence of limited amounts of lipid precursors, albeit this hypothesis needs to be further investigated.

Leucine was previously described as a precursor for sterol biosynthesis in trypanosomatids [20–22, 37]. In PCF grown in rich medium, the leucine contribution to sterol biosynthesis is 2- and 3-fold lower than glucose and acetate, respectively [22], which is consistent with our data (Fig 2). We have experimentally validated the *in silico* deduced leucine degradation pathway by demonstrating that the enzymatic activity of the *IVDH* gene product corresponds to the third enzymatic step of the pathway and that deletion of the *IVDH* gene abolished incorporation of radio-labelled leucine into sterols. The mitochondrial localization of the GFP-tagged IVDH is consistent with the potential mitochondrial targeting sequence located at the N-terminal extremity of the five enzymes potentially involved in HMG-CoA production from leucine, as well as the recent characterization of the gene encoding the mitochondrial 3-methylglutoconyl-CoA hydratase, which produces HMG-CoA from the IVDH reaction product [45]. The following step consisting on conversion of HMG-CoA into mevalonate by HMG-CoA reductase also occurs in the mitochondrial matrix [46]. This contrasts with all other eukaryotic cells, which express HMG-CoA reductase anchored to the endoplasmic reticulum membrane with its active site facing the cytosol. Thus, one may consider that the direct incorporation of leucine into sterols, unique in trypanosomatids, is the direct consequence of the unusual localization of HMG-CoA reductase in the parasite mitochondrial matrix, where HMG-CoA is produced from leucine. In other eukaryotes, HMG-CoA first needs to be converted in the mitochondrion into acetyl-CoA, which is then transferred to the cytosol through the citrate shuttle, before being converted back in the cytosol into HMG-CoA.

Our data demonstrate that the mevalonate biosynthetic pathway, leading to sterol biosynthesis, is essential for the PCF growth since the Δ*ivdh* cell line is lethal in the absence of ketogenic carbon sources, including fatty acids, and since we were not able to generate the Δ*ivdh*/^*RNAi*^SCP2 double mutant despite several attempts. This importance is certainly due to the use of mevalonate to synthesize several ubiquitous families of essential molecules such as dolichols, ubiquinones and carotenoids, that are essential for many cellular functions [47]. Mevalonate production is probably also essential for *de novo* biosynthesis of sterol, which would be consistent with previous reports showing that sterol uptake cannot compensate for lack of *de novo* synthesis [48–50]. In addition, part of *de novo*-produced ergosterol derivatives, which are not present in scavenged LDL (only cholesterol), may also serve as cell proliferation signals, as proposed for the bloodstream trypanosomes [49].

In addition to leucine, PCF trypanosomes use ketogenic carbon sources for sterol biosynthesis through conversion of acetyl-CoA into HMG-CoA by SCP2-thiolase [39] and HMGS [38], as demonstrated here by abolition of incorporation of radio-labelled glucose, threonine or acetate into sterols in the ^*RNAi*^SCP2 mutant. However, the parental cells are unable to incorporate radio-labelled leucine into fatty acids in the presence or in the absence of the main ketogenic precursors of fatty acids (glucose, threonine and acetate) (Fig 2). This implies that the 2-step bridge between the fatty acid and sterol biosynthetic pathways is unidirectional toward HMG-CoA production, even when fatty acid biosynthesis is strongly affected in the absence of ketogenic carbon sources. This observation is consistent with the 7-fold higher specific activity of *T*. *brucei* SCP2-thiolase in the synthetic direction (production of acetoacetyl-CoA from acetyl-CoA) compared to the thiolytic direction [51]. These data also suggests the absence of a HMG-CoA lyase activity (EC 4.1.3.4), converting HMG-CoA into acetoacetate and acetyl-CoA, although the *T*. *brucei* genome encodes a putative mitochondrial HMG-CoA lyase (Tb927.4.2700) [24], which was detected in the PCF proteome [52].

**Fig 10:**
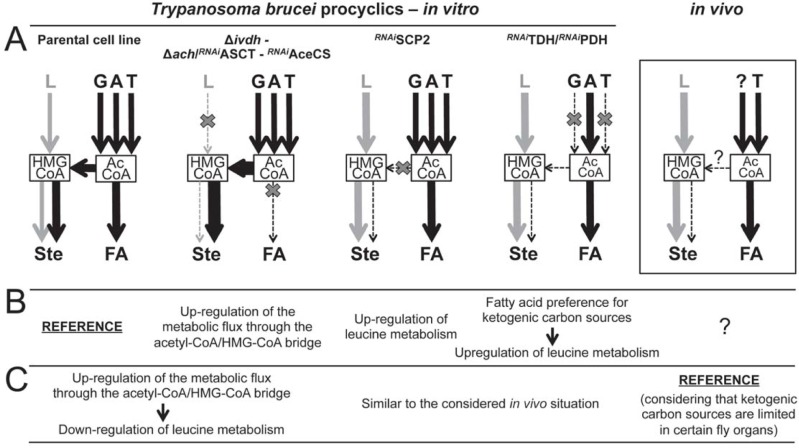
Metabolic flux distributions in parental and mutant cell lines. Schemes in panel A compare metabolic flux distribution between the different branches of fatty acid and sterol biosynthesis of the *T*. *brucei* procyclic parental and mutant cell lines grown in the carbon source-rich SDM79 medium (*in vitro*). The carbon sources included in the model are leucine, acetate, glucose and threonine, but not fatty acids, since their incorporation into lipids through *de novo* biosynthetic pathways has not been demonstrated in rich medium yet. The arrow thickness reflects the strength of metabolic flux redistributions, such as upregulation of leucine metabolism and fatty acid preference, observed in the Δ*ivdh*, Δ*ach*/^*RNAi*^ASCT, ^*RNAi*^AceCS, ^*RNAi*^SCP2 and/or ^*RNAi*^TDH/^*RNAi*^PDH mutants compared to the parental PCF cell line. The estimated flux distribution in PCF trypanosomes developing in the tsetse fly midgut is presented in the right box chart. The question mark indicates that the *in vivo* ketogenic carbon source(s) supplementing threonine, as well as the flux through the acetyl-CoA/HMG-CoA bridge are unknown; this diagram assumes a limited availability of ketogenic carbon sources. Panel B describes metabolic adaptations using as reference the parental PCF grown in rich *in vitro* conditions. The question mark means that the possible metabolic adaptation *in vivo* is still unknown, since the carbon source contents in the tsetse's organs, including the gut and salivary glands, remain unknown. In Panel C, these metabolic adaptations are re-interpreted considering the probable physiological conditions that PCF have to face *in vivo* as reference, with the assumption that ketogenic carbon sources are limited in the tsetse midgut and/or in the salivary glands. Abbreviations: A, acetate; AcCoA, acetyl-CoA; FA, fatty acids; G, glucose; HMGCoA, 3-hydroxy-3-methylglutaryl-CoA; L, leucine; T, threonine; Ste, sterols.

As discussed above, four carbon sources are used *in vitro* by PCF grown in rich medium to produce lipids, *i*.*e*. leucine only involved in sterol biosynthesis and three ketogenic sources (glucose, threonine and acetate) precursor of both sterols and fatty acids (Fig 10). It is to note that extracellular fatty acids (free fatty acids and/or phospholipids) contribute to lipid biosynthesis in the Δ*ivdh* cell lines grown in glucose/threonine/acetate-depleted conditions. However, their contribution in the wild type cells or in rich medium has not been investigated yet. Analysis of utilization of these carbon sources in mutants affecting different entry points of the *de novo* lipid biosynthetic pathways showed three kinds of flux redistribution between the mevalonate and malonyl-CoA branches. The first and most intuitive adaptation concerns up-regulation of leucine incorporation into sterols in mutants showing reduced contribution of ketogenic sources to sterol biosynthesis, such as in the ^*RNAi*^SCP2 and ^*RNAi*^TDH/^*RNAi*^PDH cell lines (Fig 10A and 10B). An equivalent flux redistribution was observed for *T*. *cruzi* epimastigotes incubated with a specific inhibitor of HMG-CoA synthase (L-659,699), which causes an increase of leucine contribution to sterol biosynthesis to compensate for blocking the “acetyl-CoA/HMG-CoA bridge” required for acetate incorporation into sterols [21]. The increase of leucine contribution to sterol biosynthesis, as a consequence of limited ketogenic source availability, is an expected adaptation to maintain the essential mevalonate pathway. The second adaptation, is the increase of threonine contribution to sterol biosynthesis in the Δ*ivdh* cell lines, which further supports the essential role of the mevalonate pathway in PCF (Fig 4C). The third type of metabolic adaptation clearly observed in the ^*RNAi*^TDH/^*RNAi*^PDH double mutant concerns shift in utilization of ketogenic carbon sources under limited carbon source degradation, *i*.*e*. when glucose and threonine contribution to acetyl-CoA production is impaired, acetate contribution to fatty acid production is increased at the expense of the abolished sterol production. Indeed, in the presence of all of the three ketogenic carbon sources at 4 mM each, 10^8^ parental PCF cells incorporate 10 and 12 nmol of acetate into fatty acids and sterols per hour, respectively (Fig 2), while the whole biosynthetic flux from acetate is redirected towards fatty acid production in the ^*RNAi*^TDH/^*RNAi*^PDH cell lines (Fig 7). This fatty acid preference would not affect the overall lipid biosynthesis, since, as mentioned above, leucine degradation can be up-regulated to fulfill the essential mevalonate demand. This adaptation is particularly relevant to sustain *de novo* biosynthesis of fatty acids by the mitochondrial FASII, which is essential for the parasite [13]. Altogether, these three levels of metabolic regulation by flux redistribution allow a high level of flexibility depending on carbon source availability to feed the mevalonate and malonyl-CoA pathways.

Although the exact composition of the tsetse midgut content is poorly known, it has been reported that this amino acid-rich environment contains at least leucine and threonine in the 100 μM range in addition to proline [53]. However, glucose is apparently absent between blood meals, and the presence of acetate and fatty acids/phospholipids has not been investigated so far. Thus, among the ketogenic carbon sources used by PCF trypanosomes, and compared to the high concentrations of all three ketogenic sources in rich cell culture conditions (4 mM each), only relatively low amounts of threonine have been detected in the tsetse midgut so far. From our *in vitro* analyses, one may consider that the possible low amounts of ketogenic carbon sources present in the insect digestive tract could be mostly, if not exclusively, used to feed fatty acid biosynthesis. In this context, leucine would be the only carbon source used by PCF in the fly midgut to feed the mevalonate pathway. Incidentally, the Δ*ivdh* mutant cell lines established fly infections as efficiently as the parental cell line, suggesting that ketogenic carbon sources feed the mevalonate pathways *in vivo*, at least when contribution of leucine to this pathway is impaired. However, we don't know yet how the IVDH null mutants would behave in the other organs of the fly. An exhaustive quantitative analysis of all possible carbon sources in the midgut, as well as in the other infected organs of the tsetse fly is now required to address this question.

In total, our observations emphasize the remarkable plasticity of the metabolic networks in this eukaryotic organism. We propose that the acetyl-CoA/HMG-CoA bridge could be used by trypanosomes to adapt to glucose/threonine/acetate-rich micro-environmental niches in the insect vector and/or in specific tissues of the mammalian hosts, by redistributing metabolic flux from ketogenic carbon sources towards the mevalonate pathway, as in the standard *in vitro* growth medium. In this context, flux through this bridge needs to be regulated as a function of carbon source availability and/or lipid requirement. Because of its key branching position, acetyl-CoA is the most promising “sentinel” candidate to relay metabolic information from this metabolic network to influence protein expression and/or activity. In favor of this hypothesis, redistribution of PCF glycolytic flux towards acetate production in the Δ*pepck* (phosphoenolpyruvate carboxykinase, EC: 4.1.1.32) mutant induced a 2-fold down-regulation of TDH expression, interpreted as a consequence of acetyl-CoA accumulation [23]. The role of acetyl-CoA in regulation of gene expression and activity through protein lysine acetylation was described decades ago. However, the intricate link between lysine acetylation and cellular metabolism is an emerging concept supported by the recent development of high throughput analyses of acetylome [54, 55]. For instance, Wang et al., showed that carbon source utilization and metabolic flux in *Salmonella* is coordinated by acetylation of key enzymes [56]. Beside histone acetylation, nothing is known about the trypanosomatid acetylome, although their genome encodes lysine acetylases and deacetylases [57–59].

## Materials and methods

### Growth and maintenance of trypanosomes

*T*. *brucei* EATRO1125.T7T (*TetR-HYG T7RNAPOL-NEO*) PCF were cultured at 27°C in SDM79 medium containing 10% (v/v) heat-inactivated FCS and 3.5 mg/l hemin [60]. Alternatively, PCF cells were cultivated into SDM79 medium depleted from glucose and/or acetate, and supplemented with 50 mM N-acetylglucosamine, a specific inhibitor of glucose transport, that prevents consumption of the fetal calf serum-derived glucose (0.5 mM final) [29, 61, 62], and with 150 μM of threonine (instead of 3.4 mM in standard SDM79) to sustain protein biosynthesis. This glucose/threonine/acetate-depleted SDM79 medium was also prepared with heat-inactivated commercial delipidated FCS (Cocalico Biologicals) that contains ~5-times less sterol and phospholipids compared to regular FCS.

### Gene knock-out and inhibition of gene expression by RNAi

Replacement of the gene encoding the PDH complex E2 subunit (PDH-E2, EC 2.3.1.12, Tb927.10.7570), ASCT (ASCT, EC 2.8.3.8, Tb927.11.2690) and ACH (ACH, EC 3.1.2.1, Tb927.3.4260) by the blasticidin (BSD) and puromycin (PAC) resistance markers via homologous recombination was described before (Δ*pdh*, Δ*asct* and Δ*ach* cell lines, respectively) [9, 23]. Replacement of the isovaleryl-CoA dehydrogenase gene (*IVDH*, EC 1.3.99.10, Tb927.11.1540) and the 2-amino-3-ketobutyrate CoA transferase gene (*AKCT*, EC 2.3.1.29, Tb927.8.6060) by BSD and PAC resistance markers via homologous recombination was performed with DNA fragments containing a resistance marker gene flanked by the IVDH or AKCT UTR sequences, as performed before [63]. Briefly, the pGEMt plasmid was used to clone a HpaI DNA fragment containing the BSD or PAC resistance marker gene preceded by the IVDH 5’UTR fragment (668 bp) and followed by the IVDH 3’UTR fragment (524 bp). For the AKCT knock-out the same procedure was performed with AKCT 5’UTR (535 bp) and AKCT 3’UTR (510 bp) fragments. The IVDH and AKCT knock-out were generated in the EATRO1125.T7T parental cell line, which constitutively expresses the T7 RNA polymerase gene and the tetracycline repressor under the control of a T7 RNA polymerase promoter for tetracycline inducible expression (*TetR-HYG T7RNAPOL-NEO*) [64]. Transfection and selection of drug-resistant clones were performed as reported previously [65]. Transfected cells were selected in SDM79 medium containing hygromycin B (25 μg/ml), neomycin (10 μg/ml), blasticidin (10 μg/ml) and puromycin (1 μg/ml). The selected cell lines *TetR-HYG T7RNAPOL-NEO Δivdh*::*BSD/Δivdh*::*PAC* and *TetR-HYG T7RNAPOL-NEO Δakct*::*BSD/Δakct*::*PAC* are called Δ*ivdh* and Δ*akct*, respectively. Re-introduction of an ectopic *IVDH* copy in one *IVDH* locus was performed by transfecting the Δ*ivdh*-C3 mutant cell line with a DNA fragment containing the IVDH 5’-UTR (346 bp) followed by the phleomycin resistant gene (*BLE*), the *aldolase* 3'UTR (146 bp), the *actin* 5'UTR (132 bp), the *IVDH* gene and the IVDH 3’UTR (362 bp). Transfected cells were selected in SDM79 medium containing hygromycin B (25 μg/ml), neomycin (10 μg/ml), blasticidin (10 μg/ml), puromycin (1 μg/ml) and phleomycin (5 μg/ml).

The inhibition by RNAi of gene expression was performed by expression of stem-loop “sense/anti-sense” RNA molecules of the targeted sequences introduced in the pLew100 (kindly provided by E. Wirtz and G. Cross) [66] or the pHD1336 (kindly provided by C. Clayton, ZMBH, Heidelberg, Germany) expression vectors, as previously described. The PCF transfected cells were selected in SDM79 containing 5 μg/ml of phleomycin (pLew100) or 10 μg/ml of blasticidin (pHD1336). Down-regulation of gene expression by RNAi of threonine dehydrogenase (TDH, EC 1.1.1.103, Tb927.6.2790) and PDH-E2 in the EATRO1125.T7T cell lines is described elsewhere (^*RNAi*^TDH and ^*RNAi*^PDH/^*RNAi*^TDH) [23]. Similarly, production of the Δ*ach*/^RNAi^ASCT cell line has been described before [9]. Construction of pLew-SCP2-SAS used to down-regulate the SCP2-thiolase (SCP2-thiolase, EC 2.3.1.9, Tb927.8.2540) by RNAi was done before [39].

### Heterologous expression of *T*. *brucei* IVDH in *E*. *coli*, protein purification and immune serum production

For heterologous expression in *E*. *coli*, the pET28 vector was used to express the *T*. *brucei* IVDH missing the N-terminal 13 amino acids, responsible for mitochondrial import. The *IVDH* gene (from position 41 to 1234 bp) containing 6 histidine codons at its 3’-extremity was inserted in the *NheI* and *BamHI* restriction sites of the pET28 vector to produce the pET28-IVDH plasmid, which was used to transform the *E*. *coli* BL21(DE3) strain harboring pGro7 plasmid (Takara Bio Inc., Japan). MZ9B culture media (1 liter) supplemented with 34 μg/ml chloramphenicol, 30 μg/ml kanamycin and 0.3 mg/ml L-arabinose (induction of the expression of GroEL-ES complex) was inoculated with 10 ml of overnight culture grown in LB medium and incubated at 37°C until the culture reached 0.6 OD600. The culture was cooled down to 20°C and the protein expression was induced by addition of IPTG to a final concentration of 0.1 mM. The growth was continued overnight at 20°C. The bacterial cells were harvested by centrifugation at 3635 g for 60 min at 4°C and suspended in sodium phosphate buffer (50 mM NaH_2_PO_4_, 0.3 M NaCl, 10% glycerol pH 8.0) supplemented with 10 mM imidazole in 0.1 g/ml ratio and stored to -70°C.

After thawing the cell suspension, lysozyme, ATP and FAD were added to a final concentration of 0.1 μg/ml, 5 mM and 50 μM, respectively. The cells were disrupted by sonication and the soluble fraction was separated by centrifuging 30,000 g for 45 min at 4°C. The supernatant was mixed with 1 ml of NiNTA beads (Qiagen) and end-over-end rotated for 30 min at 4°C. The column material was allowed to settle, the unbound fraction was collected and the beads were washed with 100 column volumes of sodium phosphate buffer with 20 mM imidazole. Bound protein was eluted with 10 column volumes of sodium phosphate buffer with 250 mM imidazole. The fractions containing the recombinant IVDH were pooled and imidazole was removed by using PD-10 column according to manufacturer’s instructions (GE Healthcare). Concentrated sample was applied to Superdex 200 HiLoad prep grade 16/60 gel-filtration column (GE Healthcare Life Sciences) equilibrated with the sodium phosphate buffer at 4°C. The purity of the sample was checked by SDS-page, showing only one prominent band. Fractions containing the IVDH were pooled, concentrated and protein concentration was determined with Nanodrop (Thermo Scientific).

The anti-IVDH immune serum was raised by Covalab in rabbit by four injections at 14-day intervals of 50 μg of IVDH recombinant nickel-purified proteins, emulsified with complete (first injection) or incomplete Freund’s adjuvant.

### Activity assay

For enzymatic activity measurements, PCF cells were washed in PBS (10’, RT, 900 g), resuspended in assay buffer and lysed by sonication (Bioruptor, Diagenode; high intensity, 5 cycles, 30sec/30sec on/off). Samples were supplemented with ‘Complete EDTA-Free’ protease-inhibitor cocktail (Roche) and the protein amount determined with the Pierce protein assay in a FLUOstar Omega plate reader. The 2-amino-3-ketobutyrate CoA transferase (AKCT) activity was measured as described before in [67]. The assay buffer contained 100 mM K-phosphate buffer pH 7.4, 2.5 mM EDTA, 0.1 mM Acetyl-CoA, 300 mM glycine, 0.1 mM 5,5'-dithiobis-(2-nitrobenzoic acid) (DTNB). The reactions were started by injection of glycine. The activity was measured in the reverse direction, *i*.*e*. the acetyl-CoA consuming direction. Freshly produced free thiol groups react with DTNB and the resulting TNB^2−^ dianion can be quantified by measuring light absorption at 405 nm. The malic enzyme activity was determined as a quality control of the cellular extracts as described before [68]. The specific activity of the isovaleryl-CoA dehydrogenase (IVDH) was measured in 100 mM KPi buffer pH 7.0 in a total volume of 0.5 ml at 25°C using a Jasco-V660 (Jasco Corporation, Japan) spectrophotometer. The reaction mixture contained 50 μM 2,6-dichloroindophenol, 1.5 mM phenazine methosulfate, 10 μM FAD and 2.6 μg of IVDH. In the reference cuvette holder 50 μM 2,6-dichloroindophenol in 100 mM KPi buffer pH 7.0 was used. The baseline reaction was measured for 3 min and the reaction was started by adding isovaleryl-CoA (52.5 μM, Sigma, ref I9381). The decrease in absorbance at 600 nm was monitored during 3 min. The conversion rate was then determined from the linear part of the reaction curve and the specific activity was calculated using the molar extinction coefficient of 20.6 mM^-1^ cm^-1^ for 2,6-dichloroindophenol [69] from four separate measurements.

### Western blot analyses

Total protein extracts of parental or mutant PCF (5x10^6^ cells) were size-fractionated by SDS-PAGE (10%) and immunoblotted on PVDF membrane (Biorad) [70]. Immunodetection was performed as described [70, 71] using as primary antibodies, the rabbit anti-SCP2-thiolase (diluted 1:50) [39], the rabbit anti-ASCT (diluted 1:100) [8], the immunopurified rabbit anti-ACH (diluted 1:10) [9], the rabbit anti-TDH (diluted 1:500) [23], the rabbit anti-GPDH (glycerol-3-phosphate dehydrogenase, Tb927.8.3530, EC 1.1.1.8; diluted 1:100) [72], the rabbit anti-ENO (enolase/2-phospho-d-glycerate hydrolase, Tb927.10.2890, EC 4.2.1.11; diluted 1:20,000) [73], the rabbit anti-IVDH (diluted 1:100), the mouse anti-PDH-E2 (diluted 1:500) [63], the mouse anti-hsp60 (diluted 1:10,000) [74] and the mouse monoclonal anti-PFR (paraflagellar rod protein 2, L8C4, 1:1000) [75], and as secondary antibodies, anti-rabbit or anti-mouse IgG conjugated to horseradish peroxidase (Sigma, 1:5000 dilution). Revelation was performed using the SuperSignal West Pico Chemiluminescent Substrate as described by the manufacturer (Thermo Scientific). Images were acquired and analyzed with the ImageQuant LAS 4000 (GE Healthcare Life Sciences).

### Expression of EGFP-tagged IVDH and TY-tagged AKCT in trypanosomes and immunofluorescence analyses

Recombinant fragments corresponding to the N-terminal extremity of *IVDH* gene, with or without the N-terminal mitochondrial signal (33 residues), followed by the enhanced green fluorescent protein (EGFP) was expressed in PCF using the pLew100 vector. PCR fragments corresponding to the first 412 amino acids of IVDH (pLew-IVDH-1/412.EGFP) and the same region deleted for the N-terminal 33 residues (pLew-ACH-34/412.EGFP) were inserted between the *XhoI* and *XbaI* restriction sites of the pLew100-EGFP1 plasmid described before [76]. For *in situ* TY1 tagging of AKCT, we used a modified pEnT6P vector kindly provided by M. Gould and M. Boshart [77]. Three PCR-amplified fragments were sequentially inserted into this vector to C-terminally tag the *AKCT* gene with a TY1 tag: the 5’ end of the downstream ORF (Tb927.8.6070) (PCR1, 221 bp) flanked by the *HindIII* (5' extremity) and *NotI*/*SpeI* (3' extremity) restriction sites, the 3’ end extremity of the *AKCT* gene without the stop codon (PCR2, 267 bp) flanked by the *NotI* and *SpeI* restriction sites, and the full length 3’UTR of *AKCT* (PCR3, 751 bp) flanked by the *BamHI* and *SphI* restriction sites. The resulting plasmid was sequenced and verified, before transfection of the EATRO1125.T7T hemizygous AKCT knock out procyclic cell line (*TetR-HYG T7RNAPOL-NEO Δakct*::*BSD*) with the *NotI*-linearized plasmid. For immunofluorescence analyses, all cell lines were fixed with 2% formaldehyde in PBS, permeabilized with 0.2% Nonidet NP-40 and spread on poly-L-lysine coated slides. After incubation for 30 min in PBS containing 3% BSA, slides were incubated with rabbit anti-ASCT (diluted 1:100) [8] and mouse anti-TY1 (BB2, diluted 1:200) [78] followed by ALEXA Fluor 594-conjugated goat anti-mouse secondary antibody (diluted 1:100) and/or ALEXA Fluor 488-conjugated goat anti-rabbit secondary antibody (diluted 1:100) (Molecular Probes). Cells were viewed with a Leica DM5500B microscope and images were captured by an ORCA-R2 camera (Hamamatsu) and Leica MM AF Imaging System software (MetaMorph). Processing was performed with ImageJ.

### Analysis of lipid labelling from radio-labelled carbon sources by HPTLC

To analyse the carbon source preference (Fig 2), 10^8^ cells in the late exponential phase were incubated for 16 h in 5 ml of modified SDM79 medium containing 4 mM of each carbon source (acetate, glucose, threonine, leucine, proline; except for isoleucine and valine, 1mM) and one radio-labelled source (10 μCi of [1-^14^C]-acetate (55.3 mCi/mmol, Perkin-Elmer, Ref NEC084), 5 μCi of D-[U-^14^C]-glucose (300 mCi/mmol, Perkin-Elmer, Ref NEC042), 2.5 μCi of L-[U-^14^C]-threonine (175 mCi/mmol, American Radiolabeled Chemicals, Ref ARC0677), 2 μCi of L-[U-^14^C]-leucine (328 mCi/mmol, Perkin-Elmer, Ref NEC279), 9 μCi of L-[U-^14^C]-proline (271 mCi/mmol, Perkin-Elmer, Ref NEC285), 1 μCi of L-[U-^14^C]-isoleucine (329 mCi/mmol, Perkin-Elmer, Ref NEC278) or 1 μCi of L-[U-^14^C]-valine (271 mCi/mmol, Perkin-Elmer, Ref NEC291). For other experiments, cells were incubated in modified SDM79 medium containing 4 mM glucose, 4 mM threonine and either 5 μCi of D-[U-^14^C]-glucose or 2.5 μCi of L-[U-^14^C]-threonine or 1 mM acetate with 10 μCi of [1-^14^C]-acetate or 1 mM leucine with 2 μCi of L-[U-^14^C]-leucine. Cells were checked microscopically for viability several times during the incubation. Subsequently, cells were spun down at 1000 g for 10 min, resuspended in 200 μl of H_2_O and lipids were extracted by 2 ml of chloroform:methanol (2:1, v/v) for 30 min at room temperature, and then washed three times with 1 ml of 0.9% NaCl. Half of the washed lipid extracts were evaporated and then dissolved in 400 μl of chloroform:methanol (2:1, v/v) (Fractions 1). The other half was also evaporated and lipids were dissolved in 1 ml of methanol/H_2_SO_4_ (40:1, v/v) for trans-esterification of the fatty acids part of lipids at 80°C for 60 min. After cooling the samples, 400 μl of hexane (99% pure) and 1.5 ml of H_2_O were added, and the mixture was homogenized vigorously during 20 sec. The samples were then centrifuged 5 min at 1-000 g to separate phases, and the hexane upper phases containing fatty acid methyl esters (FAMEs) were recovered without contact with the lower phases (Fractions 2). Aliquots (10–50 μl) of Fractions 1 and Fractions 2 (FAMEs) were then loaded onto distinct HPTLC plates (Merck) developed in hexane/ethylether/acetic acid (90:15:2, v/v) to isolate and quantify sterols (R_F_ 0.20), and FAMEs (R_F_ 0.90), respectively. Sterols and FAMEs were identified by co-migration with known standards. Their radio-labelling was then determined with a STORM 860 (GE Healthcare). For the calculation of the number of nanomoles of precursor incorporated into fatty acids or sterols, we considered that one acetyl-CoA molecule is produced per molecule of acetate, threonine and glucose consumed. When all data were normalized to the results of the parental cell line set at an arbitrary value of 100, for the PCL samples, the differences between PCL samples did not exceed ±75% of values presented in Fig 2.

### Analysis of lipid labelling from [^13^C]-enriched carbon sources by GC-MS

To characterize the *de novo* synthesized sterol and fatty acid molecules (Fig 2C), 10^8^ cells in the late exponential phase were incubated for 16 h in 5 ml of modified SDM79 medium containing 4 mM of non-enriched glucose, proline, acetate, leucine and threonine (control). For ^13^C conditions, each non-enriched carbon source was individually substituted by its ^13^C equivalent: either 4 mM of D-[U-^13^C]-glucose (Cambridge Isotope Laboratories) or 4 mM of L-[U-^13^C]-proline (Sigma-Aldrich Chemistry), 4 mM of L-[U-^13^C]-leucine (Sigma-Aldrich Chemistry), 4 mM of L-[U-^13^C]-threonine (Sigma-Aldrich Chemistry) or 4 mM of [U-^13^C]-sodium acetate (CortecNet). Cells were checked microscopically for viability several times during the incubation. Cells were then spun down at 1000 g for 10 min. A saponification step was performed, after total evaporation of the solvent, by adding 1 ml of ethanol with the internal standard α-cholestanol (5 μg) and 100 **μl** of 11 N KOH, followed by 4 h of incubation at 80°C. After the addition of 1 ml of hexane and 2 ml of water, the sterol-containing upper phase was recovered and the solvent was evaporated under an N2 gas stream. Sterols were derivatized by N,O‐bis(trimethylsilyl) trifluoroacetamide (BSTFA) for 15 min at 100°C. After complete evaporation of BSTFA under N2 gas, sterols were resuspended in 200 μl of hexane before analysis by GC-MS. GC-MS was performed using an Agilent 6850 gas chromatograph and coupled MS detector MSD 5975-EI (Agilent). An HP-5MS capillary column (5% phenyl-methyl-siloxane, 30-m, 250-mm, and 0.25-mm film thickness; Agilent) was used with helium carrier gas at 2 ml/min; injection was done in splitless mode; injector and mass spectrometry detector temperatures were set to 250°C; the oven temperature was held at 50°C for 1 min, then programmed with a 25°C/min ramp to 150°C (2-min hold) and a 10°C/min ramp to 320°C (6-min hold). Quantification of sterols was based upon peak areas that were derived from the total ion current. For determination of the stable IEF (isotope enrichment factors), the extent of ^13^C incorporation was determined by GC/MS according to the calculations reported before [79].

### NMR spectrometry analyses

10^8^ *T*. *brucei* procyclic cells were collected by centrifugation at 900 g for 10 min, washed once with PBS and incubated for 6 h at 27°C in 5 ml of incubation buffer (PBS supplemented with 5 g/l NaHCO_3_, pH 7.4), with D-[U-^13^C]-glucose (4 mM) in the presence of threonine (4 mM). The integrity of the cells during the incubation was checked by microscopic observation. Fifty microliters of maleate (20 mM) were added as internal reference to a 500 μl aliquot of the collected supernatant and ^1^H-NMR spectra were performed at 125.77 MHz on a Bruker DPX500 spectrometer equipped with a 5 mm broadband probe head. Measurements were recorded at 25°C with an ERETIC method. This method provides an electronically synthesized reference signal [80]. Acquisition conditions were as follows: 90° flip angle, 5000 Hz spectral width, 32 K memory size, and 9.3 s total recycle time. Measurements were performed with 256 scans for a total time close to 40 min. Before each experiment, the phase of the ERETIC peak was precisely adjusted. Protons linked to acetate carbon C2 generate by ^1^H-NMR five resonances, a single peak (non-enriched acetate) flanked by two doublets ([^13^C]-acetate). The central resonance (1.88 ppm) corresponding to non-enriched acetate, in the Δ*akct* mutant probably derives from an unknown internal carbon source.

### Tsetse fly maintenance, infection and dissection

Teneral males of *Glossina morsitans morsitans* from 8 to 96 hours post-eclosion were first infected through a silicone membrane with *T*. *brucei* PCF at 5 x 10^6^ parasites/ml in SDM79 culture medium. Tsetse flies were subsequently maintained in Roubaud cages for 15 days at 26°C and 60% hygrometry and fed twice a week through a silicone membrane with mechanically defibrinated fresh sheep blood as previously described [41]. Flies were starved for at least 48 hours before being dissected. Tsetse alimentary tracts from the proventriculus to the hindgut were dissected and arranged lengthways in a PBS drop. The presence of parasites was assessed by microscopic examination as previously described [41]. A total of 850 flies were dissected in three independent replicates in order to compare the infection rates obtained with the four strains.

### Statistical analyses

Tsetse fly mortality and midgut infection rates were subjected to statistical analyses with the software XLSTAT Version 2016.05.34687 (Addinsoft) integrated to Excel 15.31 (Microsoft). A two-way ANOVA test including 3 parameters (mortality rates, infection rates and number of dissected flies) was performed with intergroup comparisons by Tukey ad hoc post-tests with α = 0.05. Infection rates were plotted as mean ± SD.
